# IGF-1 in Friedreich’s Ataxia – proof-of-concept trial

**DOI:** 10.1186/2053-8871-1-10

**Published:** 2014-07-04

**Authors:** Irene Sanz-Gallego, Ignacio Torres-Aleman, Javier Arpa

**Affiliations:** Reference Unit of Hereditary Ataxias and Paraplegias, Department of Neurology, IdiPAZ, Hospital Universitario La Paz, 28046 Madrid, Spain; Neuroendocrinology Laboratory, Functional and Systems Neurobiology Department, Cajal Institute, CSIC, and CIBERNED, Avda Dr. Arce, 37, 28002 Madrid, Spain

**Keywords:** Friedreich’s ataxia, IGF-1 therapy

## Abstract

**Background:**

Friedreich’s ataxia is an autosomal recessive, severely incapacitating disorder. There is little objective evidence regarding FRDA management. Abnormalities in the insulin/insulin-like growth factor 1 (IGF-1) system (IIS) signalling pathway were thought to play a role in the physiopathological processes of various neurodegenerative disorders, including spinocerebellar ataxias. The objective of the study was to test the safety, tolerability, and efficacy of therapy with IGF-1 in Friedreich’s ataxia (FRDA) patients in a clinical pilot study.

**Results:**

A total of 4 females and 1 male were included in the study; 23 to 36 years of age (average 26.6 ± 5.4), diagnosed with FRDA with normal ventricular function. Patients were treated with IGF-1 therapy with 50 μg/kg twice a day subcutaneously for 12 months. The efficacy of this therapy was assessed by changes from baseline on the scale for the assessment and rating of ataxia, (SARA) and by changes from baseline in echocardiogram parameters. The annual worsening rate (AWR) was estimated in this series as a SARA score of -0.4 ± 0.83 (CI 95%: -1.28 to 0.48) SARA score, whereas the AWR for our FRDA cohort was estimated as a SARA score of 2.05 ± 1.23 (CI 95%: 1.58 to 2.52). Echocardiographic parameters remained normal and stable.

**Conclusion:**

Our results seem to indicate a benefit of this IGF-1 therapy to neurological functions in FRDA.

## Background

Friedreich’s ataxia (FRDA; OMIM 229300) is an autosomal recessive, severely incapacitating disorder. It involves the central and peripheral nervous system and the heart and has a major influence on the lives of affected individuals. Neurological symptoms are characterised by progressive gait instability, limb and trunk ataxia, dysarthria, decreased vibration and joint position senses, absent or reduced tendon reflexes, corticospinal tract signs, and weakness [1–3]. Patients with FRDA usually show ataxia, dysarthria and scoliosis around the time of puberty, late in the 1st decade or early in the 2nd decade (range 2 to > 70 years). Slow progression occurs with patients confined to a wheelchair after 15 years on average (ranging from a few years to decades) [4]. Life expectancy is reduced to an average of 38 years [5] (range: 21 to 69 years), with cardiomyopathy the most frequent cause of death. Only one locus has been recognised, which has been mapped to chromosome 9q13 [6]. The expanded GAA repeat results in inhibition of *FRDA* gene expression, as well as in a deficiency in *FXN* transcript levels, and ultimately in a deficiency of frataxin protein. Some 98% of patients are homozygous for GAA repeat expansions, and the remaining 2% are compound heterozygous for an expanded allele and a point mutation within the coding sequence of the gene [7, 8]. Point mutations predicting a truncated frataxin and missense mutations have been reported [6, 9–16]. Reduced frataxin expression, in turn, results in deficient assembly of iron–sulphur clusters, abnormal accumulation of intramitochondrial iron, elevated oxidative stress and impaired cellular energy production [8].

There is little objective evidence regarding FRDA management. Antioxidant therapy by free-radical scavengers including coenzyme Q_10_ and vitamin E [17–19] and idebenone (a short-chain analogue of coenzyme Q_10_) [20–24] and chelation therapy [25–27] have been considered potential treatments for slowing the progression of FRDA in some studies, but not in others [17, 28–30]. Triple therapy with darbepoetin alpha, idebenone, and riboflavin may slow FRDA disease progression [31]. The results of triple therapy with deferiprone, idebenone and riboflavin seem to indicate some uncertain benefit to neurological and heart functions in FRDA [32].

The insulin-like system plays important metabolic, trophic, and modulatory functions in the central nervous system (CNS), increasing cell proliferation, survival and antiapoptotic responses [33–35]. Abnormalities in the insulin/insulin-like growth factor 1 (IGF-1) system (IIS) signalling pathway were thought to play a role in the physiopathological processes of various neurodegenerative disorders, including Alzheimer’s disease, spinocerebellar ataxias (SCAs) and Huntington disease (HD) through various mechanisms [33, 34, 36].

These findings led to the design of the clinical pilot study described here. The primary aim of this study was to evaluate the safety and tolerability of IGF-1 therapy in patients with FRDA. The secondary objective was to evaluate the efficacy of IGF-1 therapy for the treatment of FRDA patients. A third objective was to evaluate the effect of this therapy on cardiac function.

## Results

Patients included in this study were 4 females and 1 male, 23 to 36 years of age (average 26.6 ± 5.4), diagnosed with FRDA with confirmed GAA repeat expansion mutations in the *FXN* gene and a GAA repeat ≥ 400 on the shorter allele, and disease duration of 9.4 ± 5.5 years. Each patient was treated with current, long-term Idebenone 20 mg/kg/day. Demographic and clinical variables of the 5 study patients are shown in Table 1. Patients had a baseline score between 9 and 21.5 (average 15.2 ± 4.8) on the scale for the assessment and rating of ataxia (SARA) [37]. The patients were treated with IGF-1 (mecasermin, Increlex®; Ipsen-Pharma) 50 μg/kg twice a day subcutaneously for 12 months.

**Table 1:** **Demographic and clinical characteristics of this series of FRDA patients**

Patients	Age	Gender	Disease duration	Initial SARA	GAA repeats	ECG	Initial LVMI (g/m ^2^)	IGF-1 therapy (dose)	Follow-up	Therapy withdrew	Adverse events	SF36v2:
												PC: Physical component
												MC: Mental component
**Patient 1**	23	F	12	18	>800	Widespread inferolateral T-wave inversion	Normal	50 μg/kg bid	3 four-month period	Four-month period 3	None	PC: feeling fair
												MC: feeling fair
**Patient 2**	24	F	14	21.5	>500	Widespread inferolateral T-wave inversion	Normal	50 μg/kg bid	3 four-month period	Four-month period 3	None	PC: feeling better
												MC: feeling better
**Patient 3**	26	M	4	13	>500	Widespread inferolateral T-wave inversion	Normal	50 μg/kg bid	3 four-month period	Four-month period 3	GH-secreting pituitary adenoma	PC: No change
												MC: No change
**Patient 4**	36	F	14	14.25	>500	Normal	Normal	50 μg/kg bid	3 four-month period	Ongoing	None	No change during therapy period, later worsening
**Patient 5**	24	F	3	9	>500	Widespread inferolateral T-wave inversion	Normal	50 μg/kg bid	3 four-month period	Four-month period 3	None	PC: No change
												MC: No change

### Safety and tolerability

In general, IGF-1 was well tolerated by the patients with FRDA. There were no remarkable changes from baseline in vital signs. The 26-year-old man showed an unknown GH-secreting pituitary adenoma without acromegaly, whose MRI showed an intrasellar mass at the end of study non-present in baseline MRI. However, this tumour could have already been subclinically present at the beginning of the IGF-1 treatment, because his baseline level of IGF-1 was 3.279 ng/ml (N = <2.5).

### Treatment adherence

**Figure 1:**
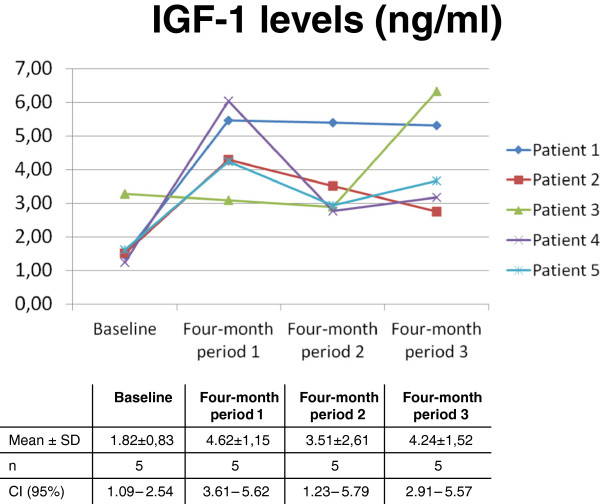
**Adherence: Significant increase in serum IGF-1 levels during the therapy period when compared with baseline.**

We also found a significant increase in serum IGF-1 levels during the therapy period when compared with baseline. Confidence interval calculated for the measure of adherence is not included within the limits of upper and lower bounds of the CI at baseline, except four-month period 2. Therefore, the adherence to IGF-1 therapy was high (Figure 1). The variations in serum IGF1 levels could be due to a variable time period between blood sample extractions and subcutaneous IGF-1 injections. Nevertheless, the IGF-1 levels during therapy were always higher than the baseline levels.

### Efficacy

#### SARA

**Figure 2:**
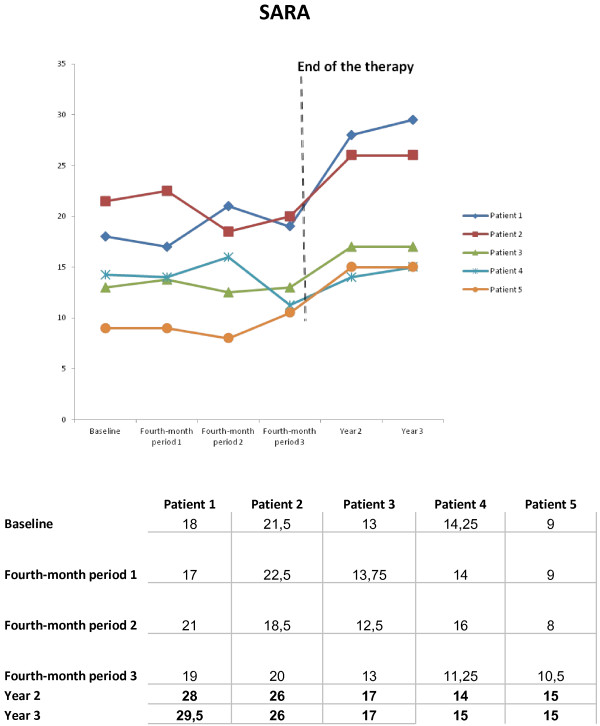
**Patients’ evolution as evaluated by the Scale for the Assessment and Rating of Ataxia (SARA).**

Statistical stabilisation of ataxia as evaluated by the SARA score was observed from the first quarter of the study (Figure 2). The annual worsening rate (AWR) was estimated in this series as -0.4 ± 0.83 (CI 95%: -1.28 to 0.48) SARA score, whereas the AWR for our FRDA cohort was estimated as a SARA score of 2.05 ± 1.23 (CI 95%: 1.58 to 2.52) (Table 2). Confidence interval calculated for the measure of treatment effect is not included within the limits of upper and lower bounds of the CI control, which would seem to indicate a decrease in the progression of the disease with IGF-1 therapy.

**Table 2:** **Annual Worsening Rate (AWR)**

FRDA patients	N	Annual worsening rate	CI 95%
Baseline – 3^rd^ four-month period (AWR)	5	-0.40 ± 0.83	-1.28 to 0.48
Control	99	2,05 ± 1,23	1,58 to 2,52

We observed a certain rebound effect with significant worsening one year after the end of the trial (*p* < 0.03), with return to the previous AWR two years after the end of the study.

Moreover, in the mixed-effects model, each individual’s vector of responses is modelled as a parametric function, whereas some of the parameters or “effects” are random variables with a multivariate normal distribution. Estimates of Fixed Effects in our series indicate that the variability in the basal score influences individual evolution (*p* < 0.01).

#### Cardiac function

Septal wall thickness (SWT), and posterior wall thickness (PWT), measured at end-diastole from M-mode recordings in a longitudinal parasternal view [38], Left Ventricular Shortening Fraction (LVSF), Left Ventricular Ejections Fraction (LVEF), and Left Ventricular Mass Index (LVMI,) of these patients were normal at baseline. At the end of the study 142 period, all of these parameters remained normal in all participants.

#### SF-36v2

Patient satisfaction with IGF-1 therapy was measured using the SF-36v2 [39]. Of the 5 patients, 40% were dissatisfied; satisfaction was fair in 60% and poor in 0% during a limited time in terms of physical and mental components of SF36v2 (Table 1).

#### Limitations of the study

This was an open-label study, with a limited number of patients (only 5), with a potentially significant initial placebo effect, and variability in the baseline scores that could have influenced individual evolution. A double-blind placebo-controlled could not be carried out because of IPSEN PHARMA, sole manufacturing and distribution’ company of IGF-1, communicated us that it has been forced to suspend, not only the production, but also the distribution of IGF-1. For which reason, we have had to cancel this kind of clinical trial that we had already approved and funded.

## Discussion

Exogenous trophic factors (GDNF and/or IGF-1) can delay the onset of hereditary Purkinje cell degeneration and gait ataxia in shaker mutant rats characterised by spatially restricted degeneration of cerebellar Purkinje neurons from adult-onset heredodegeneration [40].

Serum levels of insulin and insulin-like growth factors and their binding proteins (IGFs and IGFBPs, respectively) are changed in human neurodegenerative diseases of very different etiologies, such as Alzheimer’s disease, amyotrophic lateral sclerosis, or cerebellar ataxia [41].

Two types of late-onset cerebellar ataxias (olivopontocerebellar and idiopathic cerebellar cortical atrophy) show low IGF-I levels in the blood, high levels of IGF-binding protein 1 (IGFBP-1) and IGFBP-3 affinity for IGF-1 [42].

Both ataxic animals as well as human patients show altered serum IGF-1 levels. However, the pathogenic significance of IGF-1 in this varied group of diseases is difficult to envisage. Disrupted IGF-1 neuroprotective signalling may constitute a common stage in the pathological cascade associated with neuronal death. Treatment with IGF-1 has proven effective in neurotoxic and transgenic animal models of ataxia [43–45].

In transgenic animal models of other polyQ disorders, there was also evidence of the involvement of signalling components of the IIS in the modulation of mutant proteins and disease phenotype [46, 47].

In two mouse models of SCA1 and SCA7 that express the glutamine-expanded protein from the respective endogenous loci, transcriptional changes were found, with down-regulation of IGF binding protein 5 (*IGFBP5*) representing one of the most robust changes [48].

Two very different inherited neurodegenerative conditions, ataxia-telangiectasia (AT) and Charcot-Marie-Tooth 1A (CMT-1A) disease, serum levels of IGFs are also altered. Both types of patients have increased serum IGF-1 and IGFBP-2 levels, and decreased serum IGFBP-1 levels, while only AT patients have high serum insulin levels [41].

AT and FRDA patients, who show cumulative DNA damage, may also show disturbed IGF-1 function [49]. DNA damage is known to reduce IGF-1 activity [50].

On clinical grounds, altered serum levels of IGF-1 and IGF-1 binding proteins (IGFBPs) have been reported in patients with late onset cerebellar ataxia (LOCA) [43]. IGF-1 has therapeutic effects in various types of cerebellar ataxia [51] and exerts protective actions on mitochondrial function.

IGF-1 normalised frataxin levels in frataxin-deficient neurons and astrocytes through its canonical Akt/mTOR signalling pathway, and significantly increased levels of frataxin in cardiomyocites from conditional FRDA mouse mutants. IGF-1 normalised motor coordination in the moderately FRDA-like transgenic mice (YG8R mice) [49].

IGF-1 treatment has been tested in clinical trials for various disorders [52–54] and, with the exception of the early clinical studies, which utilised very high doses of IGF-1 that induced transient hypoglycemia, no significant adverse effect was reported.

Ventricular dysfunction was an exclusion criterion to avoid a bias in the evaluation with SARA. Since structural cardiovascular derangements are associated with an increased risk of developing heart failure including left atrial dilatation and dysregulation of breathing in chronic heart failure (CHF) might involve changes of control at several levels, ranging from peripheral ergoreflex activation and peripheral chemosensitivity, through abnormal autonomic reflexes to an altered central command, ventricular dysfunction was rule out in our patients. It has been suggested a reduction in reactivity of the cerebral circulation in CHF. Such altered reactivity might contribute to the generation of symptoms and/or the autonomic dysfunction found in CHF [55]. In consequence, the dysfunction of the nervous system induced by CHF could influence in the patient’s achievement as evaluated by the SARA score.

We have observed a certain decrease in the progression of the disease with IGF-1 therapy. However, 3 patients started to rise on SARA score about the end of the period of treatment. Hence, we think that IGF-1 cannot prevent the evolution of FRDA completely, but only significantly reduces the progression of FRDA.

The worsening observed, as rebound phenomenon after the end of the study, could be interpreted as damage from the cellular function because of withdrawal of the IGF-1 therapy.

## Conclusions

Beneficial effects seem to be observed with IGF-1 therapy in this study in terms of neurological improvement in these FRDA patients, as measured by both SARA and SF-36v2 scales. A decrease in the progression of their neurological disease was observed, together with long-term stability of the cardiac function as evaluated by echocardiographic parameters. However, we cannot assume in our cases a definitive influence on the maintenance of the heart normal function. Overall, the results of this IGF-1 therapy in our patients are better than other studies. This seems to indicate certain benefit to neurological and heart functions through this IGF-1 therapy in FRDA.

In the future, dosing could be changed from the conventional twice a day to once every 2 weeks by mean of IGF-1 microsphere therapy [56].

Further studies with more patients and double-blind placebo-controlled studies are necessary to correctly evaluate the possible effectiveness of IGF-1.

## Methods

All participants provided written informed consent to participate in this Institutional Ethics Committee on Clinical Research-approved, open-label trial. Five FRDA patients were identified from the Unit of Hereditary Ataxia and Spastic Paraplegia of the Hospital Universitario La Paz (Madrid, Spain). Before their inclusion, an echocardiographic study was carried out to rule out ventricular dysfunction, irrespective of whether or not they had left ventricular hypertrophy. The patients were treated with IGF-1 (mecasermin, Increlex®; Ipsen-Pharma) 50 μg/kg twice a day subcutaneously for 12 months. The follow up was 2 years after the end of treatment.

### Study assessments

Baseline clinical characteristics were recorded, including age, gender, height, weight, and blood pressure, medical history and medications.

The primary objective of this study was to evaluate the safety and tolerability of IGF-1 therapy in patients with FRDA. Patient safety and the tolerability of the treatment were assessed four-monthly through reports on adverse events/adverse drug reactions, serious adverse events/serious adverse drug reactions, physical examinations, ECG, results of haematology, and blood chemistry analyses. The secondary objective was to evaluate the efficacy of therapy with IGF-1 for the treatment of FRDA, as assessed by changes from baseline in SARA [37] and SF-36v2 scores [39]. The third objective was to evaluate the effect of this therapy on cardiac function, as measured by the change from baseline in SWT, and PWT, measured at end-diastole from M-mode recordings in a longitudinal parasternal view, in LVSF, LVEF, and LVMI using echocardiogram [38].

### Statistical analyses

Safety analyses were performed on the safety intent-to-treat (ITT) population, which was defined as patients who were selected and had received at least one dose of the allocated drugs. Efficacy analyses were performed on the per protocol (PP) population, defined as patients who had completed at least one year in the study and had no major protocol violations.

For the efficacy parameters, to compare the change from baseline to post-baseline visits, non-parametric Wilcoxon-Mann–Whitney tests were used.

Evolution over time was studied (quarterly) using Linear Mixed Effects Models for the adjustment of correlations caused by repeated measurements, made on the same statistical units (longitudinal study), to determine the quarterly rate of change with a confidence interval at 95%.

Previously, we had determined that in 99 patients with FRDA the mean ± SD 1-year worsening of the cohort on SARA was 2.05 ± 1.23 points.
